# Updated distribution of anopheline mosquitoes in Hokkaido, Japan, and the first evidence of *Anopheles belenrae* in Japan

**DOI:** 10.1186/s13071-021-04995-w

**Published:** 2021-09-26

**Authors:** Kyoko Sawabe, Nozomi Imanishi-Kobayashi, Yoshihide Maekawa, Yukiko Higa, Kyeong Soon Kim, Keita Hoshino, Yoshio Tsuda, Toshihiko Hayashi, Naoko Nihei, Kenji Takai, Takeshi Kurihara, Mutsuo Kobayashi

**Affiliations:** 1 Department of Medical Entomology, National Institute of Infectious Diseases, Shinjuku, Tokyo 162-8640 Japan; 2 Program for Nurturing Global Leaders in Tropical and Emerging Communicable Diseases, Graduate School of Biomedical Sciences, Nagasaki University, Nagasaki, Nagasaki 852-8523 Japan; 3 Joint Department of Veterinary Medicine, Tottori University, Tottori, Tottori 680-8553 Japan; 4 Department of Immunology and Medical Zoology, St. Marianna University School of Medicine, Kawasaki, Kanagawa 216-8511 Japan

**Keywords:** *Anopheles hyrcanus* group mosquitoes, Hokkaido Japan, ITS2 sequence

## Abstract

**Background:**

In Hokkaido, northern island of Japan, at least seven cases of falciparum malaria were reported by 1951. A survey conducted at that time was unsuccessful in implicating any mosquito species as the possible vector. Although active anopheline mosquito surveillance continued until the middle of the 1980s, there is very limited information on their current status and distribution in Japan. Therefore, this study is an update on the current status and distribution of anopheline mosquitoes in Hokkaido based on a 15-year entomological surveillance between 2001 and 2015.

**Methods:**

A survey of mosquitoes was conducted at 22 sites in Hokkaido, Japan, from 2001 to 2015. Adult mosquitoes were collected from cowsheds, lakesides, shrubs, and habitats ranging from open grassland to coniferous forest using a Centers for Disease Control and Prevention (CDC) miniature light trap enhanced with dry ice, aspirators, and sweeping nets. Larvae were collected from lakes, ponds, swamps, stagnant and flowing rivers, and paddy fields. All specimens were morphologically identified and subjected to polymerase chain reaction (PCR)-based sequence analysis of the internal transcribed spacer 2 ( ITS2) region of rDNA. Phylogenetic trees were reconstructed using the neighbor-joining method with the Kimura 2-parameter model on MEGA X version 10.2.2.

**Results:**

A total of 46 anopheline specimens were used for the phylogenetic analysis. During the survey, a new member of the *Anopheles hyrcanus* group, *An. belenrae*, was discovered in eastern Hokkaido in 2004. *Anopheles belenrae* has since then been consistently found and confirmed to inhabit only this area of Japan. Four members of the *An. hyrcanus* group, namely *An. belenrae*, *An. engarensis*, *An. lesteri*, and *An. sineroides*, have been found in Hokkaido. The results also suggest that *An. sinensis*, formerly a dominant species throughout Japan, has become a rarely found species, at least currently in Hokkaido.

**Conclusion:**

The updated distribution of anopheline mosquitoes in Hokkaido, Japan, showed considerable differences from that observed in previous surveys conducted from 1969 to 1984. In particular, areas where *An. sinensis* was previously distributed may have been greatly reduced in Hokkaido. The phylogenetic analysis revealed a novel *An. hyrcanus* group member identified as *An. belenrae*, described in South Korea in 2005. It is interesting that *An. belenrae* was confirmed to inhabit only eastern Hokkaido, Japan.

**Graphical abstract:**

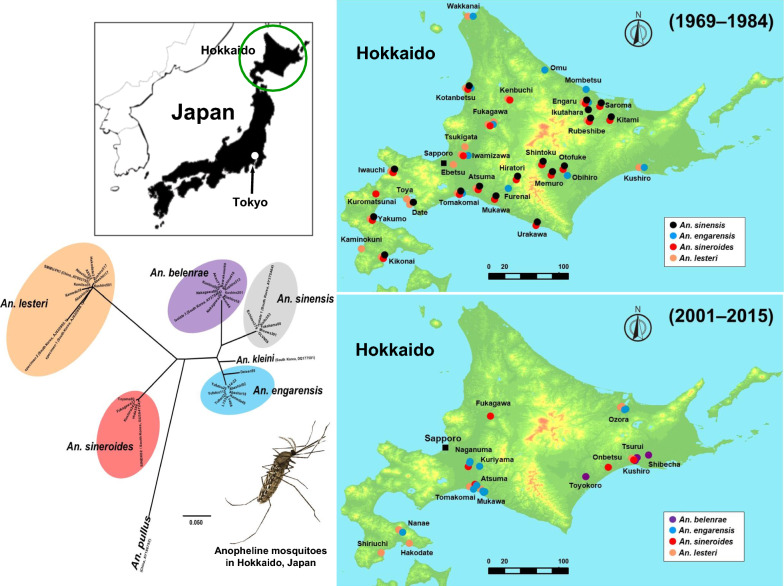

## Background

Malaria cases reported in Japan reached 28,000 annually in 1945 and 1946, with over 7000 cases of vivax malaria up to the end of the 1950s. Surprisingly, at least seven cases of falciparum malaria were reported between 1947 and 1951 in Rubeshibe, Hokkaido (43.78 N, 143.61 E), located in the north of Japan. Although a survey was conducted to determine the vector mosquitoes involved at that time, no suspected species were found [1]. Endemic malaria was considered eliminated by 1960. The number of malaria cases has decreased drastically since then, with less than 80 imported cases annually in the past 10 years, and 20 imported cases in 2020 [2].

Anopheline species contain the most important malaria vector species. Among those recorded in Japan, *Anopheles sinensis* is the most widespread and common anopheline species. This species is considered the major vector of vivax malaria in Korea and China. Previous surveys conducted in Japan from 1970 to 1986 revealed that *An. sinensis* was the dominant anopheline species in Japan, including Hokkaido; *An. lesteri* was commonly found in Hokkaido, with only a few *An. sineroides* [3–6]. These surveys also found a new member of this group, *An. engarensis* [3–5]. Thus, several malaria vector species, including *An. sinensis*, *An. engarensis*, and *An. lesteri*, continue to inhabit Japan. Despite the need for a nationwide survey to systematically assess these species, very little information is available, mostly gathered in the 1980s. Recently, several DNA barcoding projects have been conducted on mosquitoes in Japan, and a small number of genomic data on anopheline mosquitoes were included [7–9]. However, these studies were not specific to malaria vector mosquitoes.

At the onset of this survey, the presence of five species of the *An. hyrcanus* group, namely *An. sinensis*, *An. sineroides*, *An. lesteri*, *An. engarensis*, and *An. yatsushiroensis*, had been confirmed in Japan. Moreover, of these five species, only *An. yatsushiroensis* has never been reported in Hokkaido [10–13], the region of interest in this study. Nonetheless, the highly similar morphological features of the members of this group, particularly *An. engarensis* and *An. sinensis,* make it difficult to distinguish between species morphologically. Therefore, the frequency of clasper movements in males, hybridization studies, and chromosomal studies were used to distinguish *An. engarensis* from the Japanese population of *An. sinensis* [3–5]. They have recently been effectively identified using polymerase chain reaction (PCR) and sequence analysis. Among the molecular markers used for mosquito taxonomy, the cytochrome oxidase subunit 1 (*COI*) sequences of the DNA barcoding region [14–16] and the internal transcribed spacer 2 (ITS2) region of rDNA are the most efficient. ITS2 in particular is very efficient in distinguishing between closely related species such as the *An. maculipennis* complex, *An. quadrimaculatus* complex, *An. culicifacies* complex, and *An. gambiae* complex [17–20]. ITS2 has also been used to address taxonomic issues in the *An. hyrcanus* group [21–26].

For about 20 years after the last survey in 1984 [6], very few surveys of malaria vector mosquitoes were conducted in Japan. We therefore conducted nationwide surveys between 2001 and 2015 to determine the current status and distribution of anopheline mosquitoes in Japan. In the present study, species identification and determination of genetic distances between specimens was carried out by analyzing the ITS2 region. Special attention was given to determining the distribution of *An.* (*Anopheles*) *belenrae* described in South Korea in 2005 [24] in Japan. Finally, we updated the information from previous surveys [3–6] on the current distribution of the anopheline mosquitoes in Hokkaido.

## Methods

### Mosquito sampling

Hokkaido, the study site of the present survey, is generally divided into four areas: Donan (southern Hokkaido), Doo (central Hokkaido), Doto (eastern Hokkaido), and Dohoku (northern Hokkaido). Table 1 summarizes the species and numbers of mosquitoes collected from 2001 to 2015 at 22 sites in Hokkaido classified according to the above areas. The Dohoku area was not included in this survey because it is extremely mountainous, has a harsh biological environment, and does not have very convenient transportation. Anopheline mosquitoes are the most active in Hokkaido during the summer season from July to August. This period is recognized as a very short but active season for anopheline mosquitoes. Therefore, our mosquito surveys were conducted once a year in late July or August at approximately the same location.

**Table 1:** Classification results of mosquitoes collected in Hokkaido from 2001 to 2015

Area name in Hokkaido^a^		GPS coordinates		*An. belenrae*	*An. engarensis*	*An. lesteri*	*An. Sineroides*	Total no. identified	Years collected^b^
		Latitude (N)	Longitude (E)						
Doto area: Eastern Hokkaido	Ozora-1	43.8748	144.1291		9	10		19	2001 2002 2003 2004 2005 2008 2009 2010 2011 2014
	Ozora-2	43.8647	144.1082		2	7		9	
	Shibecha	43.1531	144.5072	1				1	
	Tsurui	43.1129	144.3286	1				1	
	Kushiro-1	43.0756	144.2778	1		1	1	3	
	Kushiro-2	43.0668	144.2976	17		2		19	
	Toyokoro-1	42.8206	143.5394	2				2	
	Toyokoro-2	42.8183	143.5417	1				1	
	Onbetsu	42.9644	143.8848	10				10	
Doo area: Central Hokkaido	Fukagawa	43.7499	142.0756		1		3	4	2002 2003 2014 2015
	Naganuma-1	43.0685	141.7303		1			1	
	Naganuma-2	42.9717	141.7330		2		1	3	
	Kuriyama	42.9921	141.8794		45	1		46	
	Atsuma-1	42.7002	141.8332				1	1	
	Atsuma-2	42.7000	141.8334		1			1	
	Atsuma-3	42.6407	141.7871			1		1	
	Tomakomai	42.6401	141.7883		2	2		4	
	Mukawa-1	42.5993	141.9595		69	3	1	73	
	Mukawa-2	42.5894	141.9444		1			1	
Donan area: Southern Hokkaido	Nanae	41.9792	140.6951		3	12		15	2003
	Hakodate	41.7706	140.8619			15		15	
	Shiriuchi	41.6243	140.4280			18		18	
Total no. specimens of each species				33	136	72	7	248	

^a^Hokkaido is generally divided into four areas: Doto, Doo, Donan, and Dohoku. The Dohoku area was not included in this survey. See Fig. 4 for each area in Hokkaido

^b^Mosquito surveys were conducted once a year in late July or August at approximately the same location

Adult mosquitoes were collected in cowsheds and around their habitats such as lakesides, shrubs, and open grassland to coniferous forest throughout the day using a Centers for Disease Control and Prevention (CDC) miniature light trap enhanced with dry ice [27], aspirators, and sweeping nets for approximately 3 h after sunset. Collected adult mosquitoes were frozen and transported in an icebox to the National Institute of Infectious Diseases (NIID), Tokyo, Japan. Larval mosquitoes were collected from paddy fields, swamps, stagnant and flowing rivers, lakes, and ponds using dippers. Larvae were transported alive to NIID and reared to adults under laboratory conditions of 25 °C, 60–70% relative humidity, and a photoperiod of 16:8 (L:D) h. Morphological identification was performed on all adult individuals using taxonomic keys [11, 28]. All classified mosquito specimens were transferred individually into 1.8 ml microtubes (Eppendorf, Hamburg, Germany), and stored at −80 °C until subsequent analyses by ITS2 sequencing. The collectors always wore long-sleeved shirts, long pants, and hats, and applied repellent to the bare skin of their hands and faces to prevent mosquito bites.

In this study, eight specimens collected in domestic areas outside Hokkaido were used as a reference specimen in phylogenetic analysis. Seven of the eight specimens were from Japan, and the last was from Vietnam. The areas in Japan and year surveyed were Kanagawa Prefecture in 2001, Akita Prefecture in 2005, Aomori and Toyama Prefectures in 2007, and Gifu, Fukui, and Tokushima Prefectures in 2009. The specimen collected in Gia Lai Province in Vietnam in 2007 served as an outside-Japan *An. sinensis* reference specimen. Details of the collection sites are provided in Table 2.

**Table 2:** Details of the mosquito specimens used for phylogenetic analysis in this study

Species	Specimen code	Years collected	Collection site							GenBank Accession No.	Reference
			Country	Prefecture /province	Area	GPS coordinates		Habitat (method)			
						Latitude (N)	Longitude (E)	Adult^a^	Larva		
*An. anthropophagus*	SMMU-FK1		China	Faku						AY803792	[33]
*An. belenrae*	Akan44	2004	Japan	Hokkaido	Tsurui	43.1129	144.3286	Open grassland to coniferous forest (DT)		LC634739	
	Kushiro10	2005	Japan	Hokkaido	Kushiro	43.0668	144.2976		Lake	LC634740	
	Kushiro201	2008	Japan	Hokkaido	Kushiro	43.0668	144.2976	Lakeside (SW)		LC634741	
	Kushiro313	2009	Japan	Hokkaido	Kushiro	43.0668	144.2976	Lakeside (SW)	Lake	LC634742	
	Kushiro418	2010	Japan	Hokkaido	Kushiro	43.0668	144.2976		Pond	LC634743	
	Kawakami60	2010	Japan	Hokkaido	Shibecha	43.1531	144.5072		Swamp	LC634744	
	Akan712	2011	Japan	Hokkaido	Tsurui	43.1129	144.3286		River stagnant	LC634745	
	Kushiro503	2011	Japan	Hokkaido	Kushiro	43.0756	144.2778	Cowshed (AS)		LC634746	
	Nakagawa807	2011	Japan	Hokkaido	Toyokoro	42.8206	143.5394	Cowshed (AS)		LC634747	
	Nakagawa26	2014	Japan	Hokkaido	Toyokoro	42.8183	143.5417		Swamp	LC634748	
	Isolate 3		South Korea							AY375466	[34]
*An. engarensis*	Abashiri02	2001	Japan	Hokkaido	Ozora	43.8748	144.1291	Cowshed (SW)		AB159604	
	Abashiri18	2002	Japan	Hokkaido	Ozora	43.8647	144.1082	Cowshed (SW)		LC634749	
	Yubari25	2003	Japan	Hokkaido	Kuriyama	42.9921	141.8794	Cowshed (SW)		LC634750	
	Yufutsu20	2003	Japan	Hokkaido	Mukawa	42.5993	141.9595	Cowshed (SW)		LC634751	
	Kameda40	2003	Japan	Hokkaido	Nanae	41.9792	140.6951	Cowshed (AS)		LC634752	
	Daisen55	2005	Japan	Akita	Daisen	39.5	140.425	Cowshed (AS)		LC634753	
	Yufutsu115	2014	Japan	Hokkaido	Mukawa	42.5894	141.9444	Cowshed (AS)		LC634754	
	L1532	2014	Japan	Hokkaido	Naganuma	43.0685	141.7303		Paddy field	LC634755	
	A14-22	2014	Japan	Hokkaido	Atsuma	42.7	141.8334	Shrub (DT)		LC634756	
	L1468	2014	Japan	Hokkaido	Tomakomai	42.6401	141.7883		Swamp	LC634757	
*An. kleini*			South Korea							DQ177501^b^	
*An. lesteri*	Abashiri01	2001	Japan	Hokkaido	Ozora	43.8748	144.1291	Cowshed (SW)		AB159606	
	Abashiri17	2002	Japan	Hokkaido	Ozora	43.8748	144.1291	Cowshed (SW)		LC634758	
	Abashiri42	2003	Japan	Hokkaido	Ozora	43.8647	144.1082	Cowshed (SW)		LC634759	
	Hakodate31	2003	Japan	Hokkaido	Hakodate	41.7706	140.8619	Cowshed (AS)		LC634760	
	Kamiiso35	2003	Japan	Hokkaido	Shiriuchi	41.6243	140.428	Cowshed (AS)		LC634761	
	Kameda38	2003	Japan	Hokkaido	Nanae	41.9792	140.6951	Cowshed (AS)		LC634762	
	Kushiro317	2009	Japan	Hokkaido	Kushiro	43.0668	144.2976		Lake	LC634763	
	Kushiro501	2011	Japan	Hokkaido	Kushiro	43.0756	144.2778	Cowshed (AS)		LC634764	
	A2651	2015	Japan	Hokkaido	Atsuma	42.6407	141.7871		Pond	LC634765	
	Specimen 1 (type B)		South Korea							AJ620899	[25]
	Specimen 2 (type C)		South Korea							AJ620900	[25]
*An. pullus*			China	Henan						AY186792	[22]
*An. sinensis*	Yokohama08	2001	Japan	Kanagawa	Yokohama	35.5551	139.4882	Resident (SW)		AB159603	
	Misawa391	2007	Japan	Aomori	Misawa	40.8379	141.3721	Lakeside (DT)		LC634766	
	GLVN59	2007	Vietnam	Gia Lai	Chu Se	13.6558	108.0779	Cowshed (SW)		LC634767	
	Echizen379	2009	Japan	Fukui	Echizen	35.8903	136.1961	Cowshed (DT)		LC634768	
	Kaifu353	2009	Japan	Tokushima	Kaiyo	34.0072	134.6373		Pond	LC634769	
	Isolate 1		South Korea							AY375464	[34]
*An. sineroides*	Fukagawa13	2002	Japan	Hokkaido	Fukagawa	43.7499	142.0756	Cowshed (SW)		AB159605	
	Toyama80	2007	Japan	Toyama	Toyama	36.6029	137.2682	Cowshed (DT)		LC634770	
	Kushiro343	2009	Japan	Hokkaido	Onbetsu	42.9644	143.8848		River	LC634771	
	Hida386	2009	Japan	Gifu	Hida	36.3947	137.3745	Cowshed (DT)		LC634772	
	SINEK02		South Korea							GU384724	[35]

^a^Adult mosquitoes were collected in cowsheds and around their habitats using dry-ice traps (DT), aspirators (AS), and sweeping nets (SW)

^b^ITS2 sequence was directly submitted to the GenBank database

### DNA extraction, ITS2 amplification, and sequencing

Total genomic DNA was extracted from individual samples using the REDExtract-N-Amp Tissue PCR Kit (Sigma Chemical Co., St. Louis, MO, USA) according to the manufacturer’s protocol. Extracted mosquito DNA was subjected to PCR-based sequence analysis and phylogenetic analysis using primers of the ribosomal DNA ITS2 region (forward primer, 5′-TGT GAA CTG CAG GAC ACA-3′; reverse primer, 5′-TAT GCT TAA ATT CAG GGG GT-3′) [29]. Amplification conditions were as follows: initial denaturation at 95 °C for 2 min, followed by 35 cycles of 95 °C for 30 s, 55 °C for 30 s, and 72 °C for 1 min, and a 4 min final extension at 72 °C using the Veriti™ 96-Well Thermal Cycler (Thermo Fisher Scientific Inc., Waltham, MA, USA).

All visible PCR-amplified DNA fragments were purified using the QIAquick PCR Purification Kit (QIAGEN, Venlo, Netherlands) or extracted using MonoFas (GL Sciences Inc., Tokyo, Japan) from a 2% low-melting-point agarose gel (SeaPlaque GTG Agarose, Cambrex Corp., East Rutherford, NJ, USA) after preparative gel electrophoresis and visualization with ethidium bromide. Each purified double-stranded PCR product was directly cycle-sequenced from both ends using the BigDye Terminator Cycle Sequencing FS Ready Reaction Kit v3.1 (Thermo Fisher Scientific) and the PCR primers [29]. The thermal profile used was 25 cycles of 96 °C for 10 s, 55 °C for 5 s, and 60 °C for 4 min using a thermal cycler, and an ABI PRISM 3730 Genetic Analyzer (Thermo Fisher Scientific Co.).

Sequence analysis was performed using GENETYX software version 14 (Genetyx Corp., Tokyo, Japan). Sequences of the PCR-amplified DNA fragments were then used to perform BLAST searches on the GenBank nucleic acid database of the National Center for Biotechnology Information website (http://www.ncbi.nlm.nih.gov/BLAST/) for species identification.

### Phylogenetic analysis

Multiple alignment of the ITS2 sequences with those of related species available in the GenBank library was performed using the CLUSTALW program [30]. Phylogenetic trees were produced using the neighbor-joining (NJ) program with the Kimura 2-parameter model [31] on MEGA X version 10.2.2 [32]. The statistical significance of the resulting NJ trees was evaluated using a bootstrap test with 1000 replications.

Thirty-eight specimens (37 from Japan and one from Vietnam), comprising five species, out of the total collected mosquitoes were used for phylogenetic analyses. The corresponding sequence data for eight specimens of six species, namely *An. anthropophagus* specimen “SMMU-FK1” from China (GenBank accession no. AY803792) [33], *An. belenrae* specimen “isolate 3” from South Korea (AY375466) [34], a specimen of *An. kleini* from South Korea (DQ177501, direct submission to the GenBank database), *An. lesteri* specimen “specimen 1” (type B), and specimen “specimen 2” (type C) from South Korea (AJ620899 and AJ620900, respectively) [25], *An. sinensis* specimen “isolate 1” from South Korea (AY375464) [34], and *An. sineroides* specimen “SINEK02” from South Korea (GU384724) [35], served as reference specimens. *Anopheles yatsushiroensis* from China (AY186792) [22], which was renamed *An. pullus* [36, 37], was included as an outgroup sequence. Details of all specimens analyzed in this study are shown in Table 2.

### Distribution map

A map of Hokkaido in the geodatabase (ArcGIS data collection standard pack 2014, ESRI Japan, Tokyo, Japan) was used to map the collection sites. The geographical positions of the collection sites were obtained from both previous studies [3–6] and the current study. The geographical positions obtained in this study were recorded using a geographical positioning system (GPS: GPSMAP64, Garmin, USA) (Table 1). These collection sites were plotted on the map using ArcGIS 10.0 (ESRI Inc., Redlands, CA, USA).

## Results

### Phylogenetic analysis

A total of 248 specimens (181 adults and 67 larvae) were collected in Hokkaido between 2001 and 2015. The collected specimens were classified into four anopheline species of the *An. hyrcanus* group: *An. belenrae*, *An. engarensis*, *An. lesteri*, and *An. sineroides* (Table 1). Interestingly, *An. sinensis* was not collected from Hokkaido during our survey. Phylogenetic analysis was performed using the 485-base-pair (bp) ITS2 sequence of 38 specimens, collected from different sites (30 from Hokkaido, seven from Japanese regions outside Hokkaido, and one from Vietnam) in different years, and eight reference sequences from the GenBank database (Table 2).

**Fig. 1:**
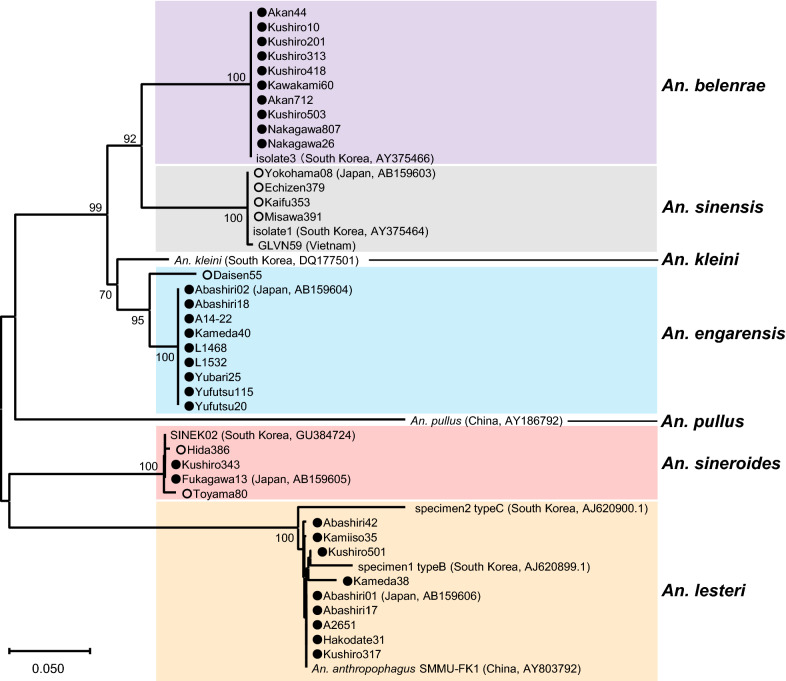
Phylogenetic relationships among members of the *Anopheles hyrcanus* group. Neighbor-joining (NJ) phylogenetic tree was constructed based on partial ITS2 region nucleotide sequences. A distance matrix was calculated using the Kimura 2-parameter evolutionary model and the tree was constructed using the NJ approach in MEGA X version 10.2.2. The scale bar indicates the proportion of sites changing along each branch. The numbers on the internodes indicate percentages of 1000 bootstrap replicates. Bootstrap values  <  70 are not shown in this figure. This phylogenetic tree is a rooted and traditional rectangular tree with *An. pullus* (AY186792) as an outgroup sequence. All specimens collected in the present study are marked with circles. The closed circles indicate mosquito specimens collected from Hokkaido, Japan, and open circles indicate specimens from the areas outside Hokkaido. Abbreviations of specimen and sequence accession numbers of specimens used in this study are listed in Table 1

**Fig. 2:**
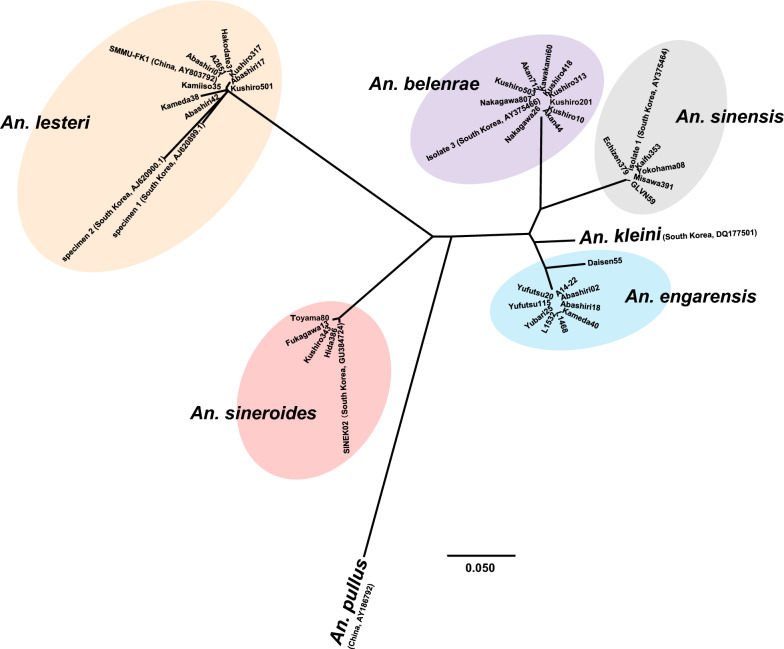
Phylogenetic relationships among members of the *Anopheles hyrcanus* group. Neighbor-joining (NJ) phylogenetic tree was constructed based on partial ITS2 region nucleotide sequences. A distance matrix was calculated using the Kimura 2parameter evolutionary model and the tree was constructed using the NJ approach in MEGA X version 10.2.2. This phylogenetic tree is a rootless and radiation tree with *An. pullus* (AY186792) as an outgroup sequence to determine more closely related species. The scale bar indicates the proportion of sites changing along each branch. Bootstrap values are not indicated in this figure. The species names in this group are in bold. The members in the same species are shaded. Abbreviations of specimen and sequence accession numbers of specimens used in this study are listed in Table 1

The NJ phylogenetic trees revealed five robust clades, consisting of the four species listed above and *An. sinensis* (Figs. 1, 2). Unfortunately, *An. sinensis* was not detected in Hokkaido in this study. Therefore, we analyzed *An. sinensis* collected from areas outside Hokkaido. The cluster of *An. sinensis* showed small differences (Figs. 1, 2). Nonetheless, there were no differences among the four Japanese specimens of *An. sinensis* (Yokohama08, Echizen379, Kaifu353, and Misawa391) and one specimen from South Korea (isolate 1).

In 2004, two larvae morphologically identified as *An. sinensis* were confirmed to be *An. belenrae* using the ITS2 sequence, marking the first record of *An. belenrae* in Japan (specimen Akan44). Subsequent phylogenetic analysis showed that *An. belenrae* was the closest related species to *An. sinensis*, followed by *An. engarensis* (Figs. 1, 2).

### Intra- and interspecific ITS2 variation

The levels of nucleotide variation detected between pairs of specimens in the *An. hyrcanus* group are presented in Table 3. There were no genetic differences between the 10 Japanese specimens of *An. belenrae* (Akan44, Kushiro10, Kushiro201, Kushiro313, Kushiro418, Kawakami60, Akan712, Kushiro503, Nakagawa807, and Nakagawa26) and the Korean strain (isolate 3), with 0% pairwise divergence. This suggests that the Japanese *An. belenrae* and the Korean *An. belenrae* are the same, at least based on the ITS2 sequences. Among the *An. sinensis* strains, the Vietnamese strain (GLVN59) showed slight differences from the other strains, with 0.26% pairwise divergence. Regarding the clusters of *An. engarensis*, one specimen (Daisen55) collected in Akita Prefecture, an area outside Hokkaido, was slightly different from the nine specimens collected in Hokkaido (Abashiri02, Akashuri18, Yubari25, Yufutsu20, Kameda40, Yufutsu115, L1532, A14-22, and L1468) (Figs. 1, 2). No genetic differences were observed among the *An. engarensis* strains, except for the specimen Daisen55, with 4.63% pairwise divergence from the others (Table 3).

**Table 3:** Percentage of pairwise divergence among five members of *Anopheles* *hyrcanus* group mosquitoes in Japan

	Specimens	1	2	3	4	5	6	7	8	9	10	11	12	13	14	15	16	17	18	19	20
1	*An. belenrae* Akan44 and 9 others, Japan																				
2	*An. belenrae* isolate 3, South Korea	***0***																			
3	*An. sinensis* Yokohama08 and 3 others, Japan	13.25	13.25																		
4	*An. sinensis* isolate 1, South Korea	13.25	13.25	***0***																	
5	*An. sinensis* GLVN59, Vietnam	13.57	13.57	***0.26***	***0.26***																
6	*An. engarensis* Abashiri02 and 8 others, Japan	13.27	13.27	13.88	13.88	14.2															
7	*An. engarensis* Daisen55, Japan	14.26	14.26	14.86	14.86	15.19	***4.63***														
8	*An. kleini*, South Korea	12.68	12.68	12	12	12.31	6.34	8.67													
9	*An. sineroides* Fukagawa13 and Kushiro343, Japan	24.58	24.58	25.32	25.32	25.71	20.94	23.76	20.98												
10	*An. sineroides* SINEK02, South Korea	24.58	24.58	25.32	25.32	25.71	20.94	23.76	20.98	***0***											
11	*An. sineroides* Hida386, Japan	24.94	24.94	25.69	25.69	26.08	21.29	24.12	21.33	***0.26***	***0.26***										
12	*An. sineroides* Toyama80, Japan	25.35	25.35	26.11	26.11	26.5	21.67	23.8	21.71	***0.8***	***0.8***	***1.06***									
13	*An. lesteri* Abashiri01 and 4 others, Japan	34.94	34.94	33.37	33.37	33.82	28.39	32.06	28.8	28.86	28.86	29.24	28.86								
14	*An. lesteri* Abashiri42, Japan	34.94	34.94	33.82	33.82	34.27	27.98	31.63	29.21	28.86	28.86	29.24	28.86	***0.26***							
15	*An. lesteri* Kameda35, Japan	34.94	34.94	33.37	33.37	33.82	28.39	32.06	28.8	28.86	28.86	29.24	28.86	***0.26***	***0.53***						
16	*An. lesteri* Kushiro501, Japan	35.35	35.35	33.77	33.77	34.22	28.77	31.67	29.18	29.24	29.24	29.62	29.24	***0.26***	***0.53***	***0.53***					
17	*An. lesteri* Kameda38, Japan	36.73	36.73	35.49	35.49	35.95	30.36	34.13	30.78	30.5	30.5	30.89	30.5	***1.87***	***2.14***	***2.14***	***2.14***				
18	*An. anthropophagus* SMMU-FK1, China	34.94	34.94	33.37	33.37	33.82	28.39	32.06	28.8	28.86	28.86	29.24	28.86	***0***	***0.26***	***0.26***	***0.26***	***1.87***			
19	*An. lesteri* specimen 1, South Korea	36.88	36.88	36.68	36.68	37.15	31.81	34.35	32.23	32.71	32.71	33.11	32.71	***2.97***	***3.24***	***3.24***	***2.69***	***4.35***	***2.97***		
20	*An. lesteri* specimen 2, South Korea	42.17	42.17	39.48	39.48	39.97	33.69	37.67	35.31	33.54	33.54	33.95	33.54	***6.9***	***7.2***	***7.2***	***7.19***	***8.67***	***6.9***	***8.96***	
21	*An. pullus*, China	38.29	38.29	38.18	38.18	37.73	34.3	33.14	33.98	37.06	37.06	37.48	36.6	41.23	41.23	41.23	40.79	42.17	41.23	43.28	49.18

The number of base substitutions per site from between sequences are shown. Analyses were conducted using the Kimura 2-parameter model [25]. This analysis involved 46 nucleotide sequences. All ambiguous positions were removed for each sequence pair (pairwise deletion option). There were a total of 485 positions in the final dataset

Evolutionary analyses were conducted in MEGA X [26]

Specimens collected in this study are labeled with the species name, specimen name, and country listed in Table 1. Specimens found in GenBank are labeled with species name, specimen name, and country

Values denoted in italic and bold indicate ITS2 intraspecific differences

As mentioned above, the intraspecific variation between these three species was very low. The pairwise divergence was 0% for *An. belenrae*, 0.26% for *An. sinensis*, and 4.63% for *An. engarensis* (Table 3). Both *An. sinensis* and *An. engarensis* indicated a few differences based on the collection areas. Regarding the interspecific variation, the pairwise divergence was 13.25–13.57% between *An. belenrae* and *An. sinensis*, 13.27–14.26% between *An. belenrae* and *An. engarensis*, and 13.86–15.19% between *An. sinensis* and *An. engarensis* (Table 3). The values among these three species indicate high levels of genetic differentiation. The NJ phylogenetic trees also showed that one specimen of *An. kleini* from South Korea was located closer to *An. engarensis* than to *An. belenrae* and *An. sinensis* (Figs. 1, 2). A detailed study of the genetic background of these species will be necessary.

In *An. sineroides*, no differences were found between the two specimens from Hokkaido (Fukagawa13 and Kushiro343) and the one from South Korea (SINEK02), with 0% pairwise divergence (Table 3). However, the two specimens from areas outside Hokkaido (Hida386 and Toyama80) showed a few differences from the three mentioned above, with 0.26 and 0.8% pairwise divergence (Table 3). In contrast, a large intraspecific variation was observed in *An. lesteri* (Figs. 1, 2). Pairwise divergence was in the range of 0.26–2.14% among the nine specimens from Hokkaido (Abashiri01, Abashiri17, Abashiri42, Hakodate31, Kamiiso35, Kameda38, Kushiro317, Kushiro505, and A2651) (Table 3). The two Korean strains (specimen 1 and specimen 2 classified as type B and type C of *An. lesteri*, respectively) were quite distant from the other *An. lesteri* strains (Figs. 1, 2). Pairwise divergence among the 11 *An. lesteri* specimens was 0.26–8.96%, indicating that *An. lesteri* appeared to form a highly divergent population. The cluster of *An. lesteri* revealed low pairwise divergence, ranging from 0 to 2.14%, between the nine *An. lesteri* specimens from Japan and the Chinese strain of *An. anthropophagus* (SMMU-FK1) (Table 3), suggesting that they may belong to the same species.

## Discussion

### The first record of *An. belenrae* in Japan

Our surveys from 2001 to 2015 revealed a significant change in the distribution range of the *An. hyrcanus* group in Hokkaido reported until the 1980s [3–6], including the first record of *An. belenrae* in Japan. Two larvae collected in the Kushiro Wetland in 2004 were tentatively named *An. sinensis* Kushiro strain, based solely on the morphological characteristics of the emerged adults. However, phylogenetic trees constructed using ITS2 sequence revealed that this *An. sinensis* Kushiro strain formed a robust clade that was clearly different from the clades of *An. sinensis* and other *Anopheles* species. Interestingly, the ITS2 sequence of the Kushiro strain was not identical to that of the *An. sinensis* strain collected in southern Japan, outside Hokkaido, but to that of *An. belenrae*, a new strain reported in South Korea in 2005 [24]. The Kushiro strain could confidently be included in the *An. belenrae* cluster because of the absence of intraspecific divergence as mentioned above. This species was consistently found in the Kushiro Wetland after the first detection in 2004. In contrast, *An. belenrae* was not found outside Hokkaido in our 15-year nationwide survey. Thus, we concluded that this species is restricted to the Kushiro Wetland in Hokkaido.

The Kushiro Wetland is the largest marshland/wetland in Japan and is located in the Kushiro Plain. The Kushiro Wetland has been the focus of nature conservation efforts since before World War II, was registered as a Ramsar site in 1980, and designated as a national park in 1987. It is also famous for being the breeding ground for Japanese cranes, *Grus japonensis*, and many other wild birds and a protected area for natural monuments, birds, and animals; thus, land development is strictly regulated. In South Korea, *An. belenrae* is found in the northern part of the country near the border with North Korea [24, 38, 39]. In China, *An. belenrae* is reportedly distributed in Shandong and Liaoning Provinces in northeastern China, facing the Korean Peninsula [40]. These areas are not only geographically close to Japan, but may also have similarities in climate, vegetation, and some environmental factors with the Kushiro Wetland. However, further investigation is needed to compare the morphological characteristics of Japanese and Korean *An. belenrae*, and to determine the distribution of this species in locations outside Hokkaido in Japan. We hope that ecological and evolutionary factors impacting the emergence of *An. belenrae* will be elucidated with the development of molecular biological technology.

### No information for *An. sinensis* from Hokkaido

**Fig. 3:**
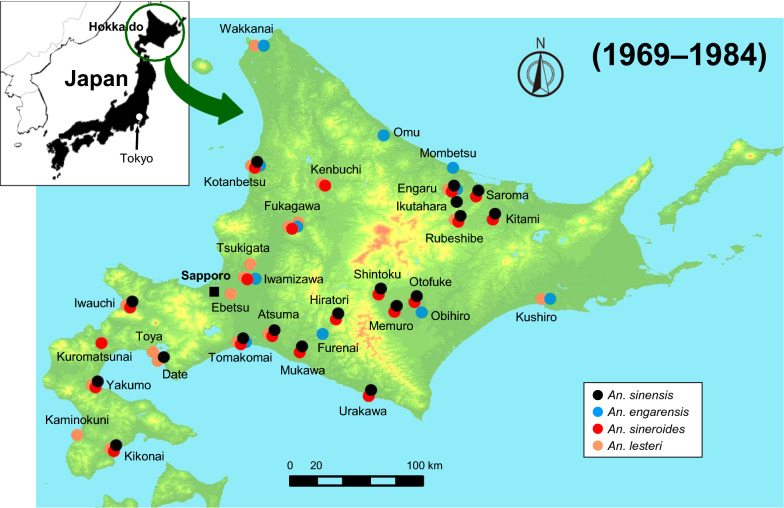
Distribution map of the *Anopheles hyrcanus* group mosquitoes in Hokkaido, Japan, as confirmed by surveys conducted during 1969–1984. This map was created from references [3–6]. This map shows mosquito species collected from 29 sites. Black, blue, red, and orange circles indicate collection sites of *An. sinensis, An. engarensis*, *An. sineroides*, and *An. lesteri*, respectively

**Fig. 4:**
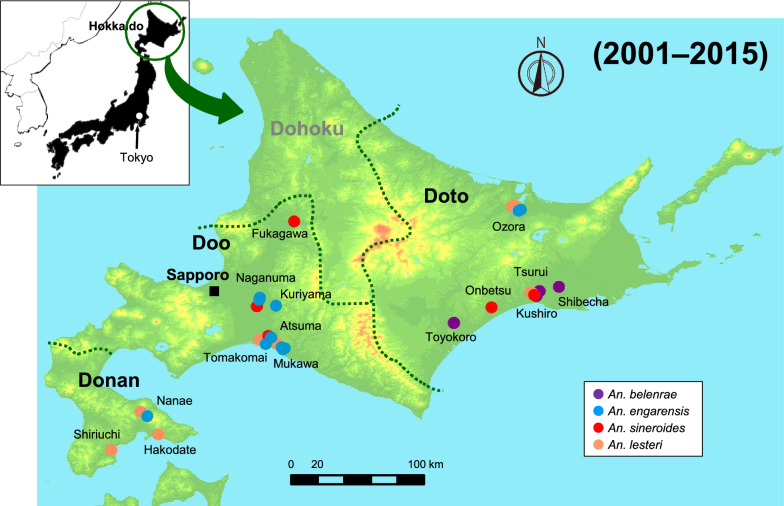
Distribution map of the *Anopheles hyrcanus* group mosquitoes in Hokkaido, Japan, as disclosed by the present study conducted during 2001–2015. This map shows mosquito species collected from 15 collection sites. Purple, blue, red, and orange circles show collection sites of *An. belenrae*, *An. engarensis*, *An. sineroides*, and *An. lesteri*, respectively

The next noteworthy finding was the disappearance of *An. sinensis* from Hokkaido. In previous surveys, *An. sinensis* was generally distributed throughout Hokkaido [3–6] (Fig. 3). Although it is often found in the same larval habitat as *An. lesteri*, it is thought to occur more frequently in developed paddy fields and swamps [41]. In the 2000s, we did not find any *An. sinensis* in the habitat of *An. lesteri*, nor did we find any new sources or habitats (Fig. 4). It is possible that the larval habitat of *An. sinensis* changed drastically during the 20-year period between the previous studies [3–6] and this current study. For example, in the 1949 [1] and 1976 [6] surveys, four members of the *An. hyrcanus* group were detected in northeastern Hokkaido, around Rubeshibe (Fig. 3). At that time, there were paddy fields all over the district, and forestry and horse-logging were the main industries. In recent times, however, the horse-logging industry has declined dramatically, and the paddy fields have been replaced with upland crops. Furthermore, neither *An. sinensis* nor *An. sineroides* was found in this area, around Ozora, during our survey (Fig. 4). It is highly likely that the changes in vegetation and industry have affected the distribution of anopheline mosquitoes.

There may be other reasons for the disappearance of *An. sinensis* from Hokkaido. The classification of organisms was mainly based on morphological keys until the 1990s. Although adults of *An. belenrae* can be separated morphologically from those of *An. lesteri*, *An. sinensis* and other species [24], in fact, it is likely that *An. belenrae* and *An. sinensis* could not be differentiated morphologically. Therefore, it should be noted that *An. belenrae* may have been classified as *An. sinensis*. The results from the ITS2 sequences in this study revealed that these two species were genetically the most closely related. In addition, the pairwise interspecific distance in mitochondrial genomes calculated by each fragment showed minor or no differences between *An. sinensis*, *An. belenrae*, and *An. kleini* [40]. Phylogenetic analysis of *COI* indicated that ancient hybridizations probably occurred among these three closely related species [42], making differentiation with the *COI* sequence improbable. To address this problem, we tried to extract DNA and decipher the nucleotide sequence from age-old, dried specimens previously classified as *An. sinensis* collected in Hokkaido [3–6]. However, no new information could be obtained from these specimens. We hope that techniques for genetic analysis using age-old specimens will soon be available.

### *Anopheles engarensis* in Japan

*Anopheles engarensis* is also a species whose distributional range in Hokkaido has decreased. This species, first described in Engaro-cho (northeastern Hokkaido) in 1977 [3], was also found in Monbetsu, Kushiro, and Obihiro until 1984 [6], suggesting a wide distribution in Hokkaido [3–6] (Fig. 3). However, our surveillance found this species to be restricted to western and southern Hokkaido (Fig. 4). In addition, the species was collected in northern Tohoku, Akita Prefecture, suggesting a southward shift presumably due to environmental changes, including the climate of larval habitats. In terms of classification, *An. engarensis* was recognized as a new species in the *An. hyrcanus* group only after its chromosomal structure was determined to be different from that of *An. sinensis* [4]. This was because of the high morphological similarity between the two species. Indeed, the only distinguishing feature was the unique number of clasper movements of *An. engarensis* males during artificial mating, a common method for laboratory maintenance of anopheline mosquitoes [5].

In general, ITS2 is known to have high interspecific and low intraspecific variability; however, extensive intraspecific variations have been reported in anopheline mosquitoes. For instance, ITS2 intraspecific variations ranged from 0.2 to 19.0% for the Latin American anophelines [43]. In the *An. hyrcanus* group, the average intraspecific distance was 0.3%, but no intraspecific variations were observed in *An. belenrae* [42]. These results suggest that the ITS2 spacer is a good marker for differentiating between members of the *An. hyrcanus* group. In this study, there were no intraspecific variations in the *An. belenrae*, *An. engarensis*, and *An. sineroides* strains from Hokkaido. However, there was significant intraspecific variation among the nine *An. engarensis* strains from Hokkaido and the specimen Daisen55 from Akita Prefecture. The genetic distance of 4.7% was considerably greater than the 0.22% intraspecific variation in the *An. sinensis* strains from Vietnam, South Korea, and Japan. We inferred that the Daisen55 *An. engarensis* strain was not introduced from Hokkaido but inhabited the Tohoku region independently. On the other hand, in species groups consisting of recently diverged members, such as the *An. gambiae* complex, the interspecific differences in ITS2 were reported to be minor, ranging from 0.4 to 1.6% [20]. It is possible that *An. engarensis* is a recently diverged lineage.

### *Anopheles lesteri* in Hokkaido

In Rubeshibe Hokkaido, at least seven cases of falciparum malaria were recorded between 1946 and 1947. A survey conducted to determine the vector mosquitoes involved in the transmission was unsuccessful, although *An. sinensis* and *An. sineroides* were collected [1]. During the falciparum malaria epidemic in the vicinity of Guangdong City, China, around 1942, the transmission was inferred to have involved *An. lesteri* and not *An. sinensis*. This inference was based on results from field investigations and subsequent infection experiments with *Plasmodium falciparum* [44]. Based on this inference, it was suggested but never confirmed that *An. lesteri* may have been involved in the outbreak of falciparum malaria in Rubeshibe, Hokkaido. In terms of distribution, *An. lesteri*, which was initially thought to be restricted to western islands of Japan such as the Kyushu Island [44], was also found in various areas of Honshu in the mainland of Japan [12], Hokkaido [45], Okinawa Island, and Yaeyama Islands [46]. The present survey confirmed that *An. lesteri* is still widely distributed in Hokkaido (Figs. 3, 4). At the start of our survey in 2001, we observed female mosquitoes collected in Ozora, Hokkaido, to have an intense affinity for human blood. These female mosquitoes were therefore considered, and subsequently confirmed, to be *An. lesteri* based on the reported high anthropophilic nature of *An. lesteri* relative to *An. sinensis* and other members of the *An. hyrcanus* group [12, 47]. Consequently, we expected to easily collect *An. lesteri* in subsequent surveys in Hokkaido.

In our study, the ITS2 intraspecific divergence in *An. lesteri* was 0–9.44%. These values suggest that *An. lesteri* is a highly divergent species when *An. lesteri* type B (specimen 1) and type C (specimen 2) from South Korea are included in this species. Since the ITS2 distance of this species varies even within Hokkaido, there is a possibility that *An. lesteri* includes crypto-species. In defining this species, it is necessary to analyze both the *COI* barcoding region and the ITS2 region. Moreover, a large number of specimens collected outside Hokkaido will be necessary. In a previous study, a short interspecific divergence of 7.2% was observed between *An. kleini* and *An. engarensis* [42]. We obtained similar ITS2 divergence of 6.47% and 8.67% between *An. kleini* and our *An. engarensis* specimens from Hokkaido and *An. kleini* and *An. engarensis* specimen Daisen55, respectively. Although these results may provide validation that *An. kleini* is a synonym of *An. engarensis*, further analysis is required. We also presented evidence that *An. anthropophagus* and *An. lesteri* were conspecific, based on the ITS2 divergence between them. Our results based on interspecific comparisons of ITS2 divergence may also support previous reports that *An. belenrae* and *An. sinensis* are genetically distinct [24, 25], and *An. anthropophagus* is a conspecific species of *An. lesteri* [34, 48].

### Changes in the distribution of the *An. hyrcanus* group in Hokkaido

In previous studies, *An. sinensis*, *An. lesteri*, and *An. yatsushiroensis* in Japan were reported to preferentially invade livestock barns and houses [12]. These species are considered to be more endophilic. However, there is very limited information on the behavior of other members of the *An. hyrcanus* group. Therefore, in order to collect as many mosquitoes as possible in this study, we attempted to collect both adults and larvae of mosquitoes from all areas using several methods regardless of mosquito behavior. In addition, the surveys were conducted in late July and August, when the mosquitoes are the most active in Hokkaido. In the previous studies [3–6], most specimens were obtained in August as well. The selection of the present survey sites was also based on these precedents. As we did not plan to conduct regular annual surveys, some negative factors such as the small number of surveys and collection sites may be considered as limitations. However, at least at the time of our survey, *An. sinensis* may not have been distributed in Hokkaido or may no longer have been present in sufficient numbers to be collected. In fact, the *An. sinensis* specimens used in this study were collected easily in areas south of Hokkaido, such as Kanagawa, Aomori, Fukui, and Tokushima Prefectures, using the same method as that followed in Hokkaido. Therefore, it is unlikely that the sampling methodology or the timing of the survey influenced our results. Similarly, our results indicate a significant change in the distribution range of other members of the *An. hyrcanus* group in Hokkaido from that reported until the 1980s [3–6]. However, although multiple surveys were conducted in the Doo and Doto areas, no further information is available for the Donan area, except for the results of a survey conducted in 2003. The possibility that the distribution of *An. sinensis* was reconfirmed after 2003 cannot be ruled out. To address these questions, continued mosquito surveys are needed in the future.

## Conclusions

ITS2 sequence divergence revealed the current distribution of the *An. hyrcanus* group mosquitoes in Hokkaido, demonstrating great differences from surveys conducted between 1969 and 1984. In particular, the area inhabited by *An. sinensis* has greatly diminished, and the newly discovered *An. belenrae* was confirmed to inhabit only eastern Hokkaido. In summary, this study showed that Hokkaido harbored four members of the *An. hyrcanus* group, namely *An. engarensis*, *An. belenrae, An. sineroides,* and *An. lesteri*.

Our research has revealed that two anopheline species reported as malaria vectors, *An. lesteri* and *An. belenrae*, are present in Hokkaido today. Although the malaria vector capacity of the Japanese strain of *An. belenrae* has not yet been evaluated, the Korean strain is considered to be a vector or potential vector of *Plasmodium vivax* [24, 49]. Fortunately, all recently reported cases of malaria in Japan have been imported. However, emergence of potential autochthonous malaria epidemics should always be of concern, because multiple malaria vector species still remain in Japan, as confirmed in this study.

## Data Availability

All data generated or analyzed during this study are included in this published article. The newly generated sequences were submitted in the GenBank database under the accession numbers LC634739‒LC634772.

## Abbreviations

NIID: National Institute of Infectious Diseases
ITS2: Internal transcribed spacer 2
*COI*: Cytochrome oxidase subunit 1

## References

[1] Sasa M, Takahashi H, Kohgo T, Oshima H (1949). An epidemic of the falciparum malaria in Hokkaido. Sogo Igaku.

[2] Infectious Diseases Weekly Report (IDWR), IDWR surveillance data table 2020 week 53. National Institute of Infectious Diseases, Japan. 2020. https://www.niid.go.jp/niid/ja/data/10103-idwr-sokuho-data-j-2053.html. Accessed 01 May 2021.

[3] Oguma M, Kanda T (1977). The distribution of *Anopheles**sinensis*, *A.**sinensis* “E” *A.**lesteri* and *A.**sineroides* at thirty-four localities of Japan. Jpn J Sanit Zool.

[4] Kanda T, Oguma Y (1978). *Anopheles engarensis*, a new species related to *sinensis* from Hokkaido Island. Japan Mosg Syst.

[5] Kanda T, Oguma Y (1976). Morphological variations of *Anopheles**sinensis* Wiedemann, 1828 and *A.**lesteri* Baisas and Hu, 1936 and frequency of clasper movements of the males of several *Anopheles* species during induced copulation. Jpn J Sanit Zool.

[6] Nagashima Y (1986). The research for *Anopheles* (*Anopheles*) *sinensis* group with reference to epidemiological aspects of malaria in Japan. Niigata Med J.

[7] Taira K, Toma T, Tamashiro M, Miyagi I (2012). DNA barcoding for identication of mosquitoes (Diptera: Culicidae) from the Ryukyu Archipelago. Japan Med Entomol Zool.

[8] Maekawa Y, Tsuda Y, Sawabe K (2016). A nationwide survey on distribution of mosquitoes in Japan. Med Entomol Zool.

[9] Maekawa Y, Ogawa K, Komagata O, Tsuda Y, Sawabe K (2016). DNA barcording for molecular identification of Japanese mosquitoes. Med Entomol Zool.

[10] Reid JA (1953). The *Anopheles**hyrcanus* group in South-East Asia. Bull Ent Res.

[11] Tanaka K, Mizusawa K, Saugustad ES (1979). A revision of the adult and larval mosquitoes of Japan (including the Ryukyu archipelago and the Ogasawara Islands) and Korea (Diptera: Culicidae). Contr Am Entomol Inst.

[12] Otsuru M, Ohmori N (1960). Malaria studies in Japan after World War II. Part II. The research for *Anopheles**sinensis* sibling species group. Jpn J Exp Med.

[13] Harrison BA (1973). A lectotype designation and description for *Anopheles* (*An.*) *sinensis* Wiedemann l828, with a discussion of the classification and vector status of this and some other oriental *Anoρheles*. Mosg Syst.

[14] Folmer O, Black M, Hoeh W, Lutz R, Vrijenhoek R (1994). DNA primers for amplication of mitochondrial cytochrome coxidase subunit I from diverse metazoan invertebrates. Mol Mar Biol Biotechnol.

[15] Hebert PD, Cywinska A, Ball SL, Waard JR (2003). Biological identifications through DNA barcodes. Proc Biol Sci.

[16] Hebert PD, Penton EH, Burns JM, Janzen DH, Hallwachs W (2004). Ten species in one: DNA barcoding reveals cryptic species in the neotropical skipper butterfly *Astraptes fulgerator*. Proc Natl Acad Sci USA.

[17] Proft J, Maier WA, Kampen H (1999). Identification of six sibling species of the *Anopheles maculipennis* complex (Diptera: Culicidae) by a polymerase chain reaction assay. Parasitol Res.

[18] Cornel AJ, Porter CH, Collins FH (1996). Polymerase chain reaction species diagnostic assay for *Anopheles quadrimaculatus* cryptic species (Diptera: Culicidae) based on ribosomal DNA ITS2 sequences. J Med Entomol.

[19] Goswami G, Raghavendra K, Nanda N, Gakhar SK, Subbarao SK (2005). PCR-RFLP of mitochondrial cytochrome oxidase subunit II and ITS2 of ribosomal DNA: markers for the identification of members of the *Anopheles culicifacies* complex (Diptera: Culicidae). Acta Trop.

[20] Paskewitz SM, Wesson DM, Collins FH (1993). The internal transcribed spacers of ribosomal DNA in five members of the *Anopheles gambiae* species complex. Insect Mol Biol.

[21] Gao Q, Beebe NW, Cooper RD (2004). Molecular identification of the malaria vectors *Anopheles anthropophagus* and *Anopheles sinensis* (Diptera: Culicidae) in central China using polymerase chain reaction and appraisal of their position within the *hyrcanus* group. J Med Entomol.

[22] Yajun M, Xu J (2005). The *hyrcanus* group of *Anopheles* (*Anopheles*) in China (Diptera: Culicidae): species discrimination and phylogenetic relationships inferred by ribosomal DNA internal transcribed spacer 2 sequences. J Med Entomol.

[23] Li C, Lee JS, Groebner JL, Kim HC, Klein TA, O’Guinn ML, Wilkerson RC (2005). A newly recognized species in the *Anopheles**hyrcanus* group and molecular identification of related species from the Republic of South Korea (Diptera: Culicidae). Zootaxa.

[24] Rueda LM (2005). Two new species of *Anopheles* (*Anopheles*) *hyrcanus* group (Diptera: Culicidae) from the Republic of South Korea. Zootaxa.

[25] Hwang UW (2007). Revisited ITS2 phylogeny of *Anopheles* (*Anopheles*) *hyrcanus* group mosquitoes: reexamination of unidentified and misidentified ITS2 sequences. Parasitol Res.

[26] Djadid ND, Jazayeri H, Gholizadeh S, Rad SP, Zakeri S (2009). First record of a new member of *Anopheles**hyrcanus* group from Iran: molecular identification, diagnosis, phylogeny, status of kdr resistance and *Plasmodium infection*. J Med Entomol.

[27] Tsuda Y, Higa Y, Kurahashi H, Hayashi T, Hoshino K, Komagata O, Isawa H, Kasai S, Sasaki T, Tomita T, Sawabe K, Nihei N, Kobayashi M (2006). Dry-ice trap collection of mosquitoes at urban areas surrounding Tokyo, Japan in 2003 and 2004. Med Entomol Zool.

[28] Stojanovich CJ, Scott HG (1966). Illustrated key to mosquitoes of Vietnam.

[29] Beebe NW, Saul A (1995). Discrimination of all members of the *Anopheles punctulatus* complex by polymerase chain reaction-restriction fragment length polymorphism analysis. Am J Trop Med Hyg.

[30] Thompson JD, Higgins DG, Gibson TJ (1994). CLUSTAL W: improving the sensitivity of progressive multiple sequence alignment through sequence weighting, position-specific gap penalties and weight matrix choice. Nucleic Acids Res.

[31] Kimura M (1980). A simple method for estimating evolutionary rates of base substitutions through comparative studies of nucleotide sequences. J Mol Evol.

[32] Kumar S, Stecher G, Li M, Knyaz C, Tamura K (2018). MEGA X: molecular evolutionary genetics analysis across computing platforms. Mol Biol Evol.

[33] Ma Y, Yang P (2005). Taxonomic study on *Anopheles anthropophagus* from China (Diptera: Culicidae): inferred by morphology, chromosome karyotype and molecular markers. Kun Chong Fen Lei Xue Bao.

[34] Wilkerson RC, Li C, Rueda LM, Kim HC, Klein TA, Song GH, Strickman D (2003). Molecular confirmation of *Anopheles* (*Anopheles*) *lesteri* from the Republic of South Korea and its genetic identity with *An.* (*Ano*) anthropophagus from China (Diptera: Culicidae). Zootaxa.

[35] Joshi D, Park MH, Saeung A, Choochote W, Min GS (2010). Multiplex assay to identify Korean vectors of malaria. Mol Ecol Resour.

[36] Shin E, Hong HK (2001). A new synonym of *Anopheles* (*Anopheles*) *pullus* Yamada, 1937: *A.* (*A.*) *yatsushiroensis* Miyazaki, 1951. Korean J Appl Entomol.

[37] Hwang UW, Yong TS, Ree H (2004). Molecular evidence for synonymy of *Anopheles**yatsushiroensis* and *An.**pullus*. J Am Mosq Control Assoc.

[38] Rueda LM, Kim HC, Klein TA, Pecor JE, Li C, Sithiprasasna R, Debbouns M, Wilkerson RC (2006). Distribution and larval habitat characteristics of *Anopheles**hyrcanus* group and related mosquito species (Diptera: Culicidae) in South Korea. J Vector Ecol.

[39] Kim HC, Klein TA, Lee WJ (2007). Mosquito species distribution and larval breeding habitats with taxonomic identification of anopheline mosquitoes in Korea. Entomol Res.

[40] Zhu HM, Luo SH, Gao M, Tao F, Gao JP, Chen HM, Li XY, Peng H, Ma YJ (2019). Phylogeny of certain members of *hyrcanus* group (Diptera: Culicidae) in China based on mitochondrial genome fragments. Infect Dis Poverty.

[41] Kurihara T (2002). Malaria vectors in Japan (except Nansei Islands). Med Entomol Zool.

[42] Fang Y, Shi WQ, Zhang Y (2017). Molecular phylogeny of *Anopheles hyrcanus* group members based on ITS2 rDNA. Parasites Vectors.

[43] Marrelli MT, Sallum MAM, Marinotti O (2006). The second internal transcribed spacer of nuclear ribosomal DNA as a tool for Latin American anopheline taxonomy—a critical review. Mem Inst Oswaldo Cruz.

[44] Otsuru M (1949). On a new type of *Anopheles* hyrcan from Japan. Fukuoka Med J.

[45] Kamimura K (1968). The distribution and habit of medically important mosquitoes of Japan. Med Entomol Zool.

[46] Tanaka K (1975). Mosquitoes of the Ryukyu archipelago (Diptera: Culicidae). Mosq Syst.

[47] Ho C, Chou TC, Chen TH, Hseuh AT (1962). The *Anopheles* hyrcanus group and its relation to malaria in East China. Chin Med J.

[48] Hwang UW, Tang LH, Kobayashi M, Yong TS, Ree HII (2006). Molecular evidence supports that *Anopheles anthropophagus* from China and *Anopheles lesteri* from Japan are the same species. J Am Mosq Control Assoc.

[49] Rueda LM, Li C, Kim HC, Klein TA, Foley DH, Wilkerson RC (2010). *Anopheles belenrae*, a potential vector of *Plasmodium vivax* in the Republic of Korea. J Am Mosq Contol Assoc.

